# Development and treatment of steroid resistant asthma model by adoptive transfer of murine helper t cell clones

**DOI:** 10.1186/1939-4551-8-S1-A3

**Published:** 2015-04-08

**Authors:** Akio Mori, Satoshi Kouyama, Miyako Yamaguchi, Yo Iijima, Akemi Ohtomo, Takayuki Ohtomo, Jun Itoh, Kentaro Watarai, Chihiro Mitsui, Chiyako Oshikata, Yuma Fukutomi, Kiyoshi Sekiya, Hiroaki Hayashi, Takahiro Tsuburai, Yuji Maeda

**Affiliations:** 1 National Hospital Organization, Sagamihara National Hospital, Japan

## Background

To investigate the role of helper T (Th) cells in steroid resistant (SR) asthma, steroid sensitive (SS) and resistant (SR) Th clones were selected *in vitro*, and then adoptively transferred into unprimed mice. Effect of CTLA4-Ig was analyzed both *in vitro* and *in vivo*.

## Methods

For *in vitro* evaluation of steroid sensitivity, ovalbumin (OVA) reactive Th clones were cultured with antigen presenting cells and OVA in the presence of various concentrations of dexamethasone (DEX). Proliferative responses of Th clones were measured by ^3^H-thymidine incorporation. For *in vivo* evaluation, unprimed BALB/c mice were transferred with Th clones, challenged with OVA, and administered with DEX subcutaneously. Bronchoalveolar lavage fluid (BALF) was obtained 48 hr after challenge, and the number of infiltrating cells was differentially counted. CTLA4-Ig was administered either intravenously or intranasally.

## Results

SS and SR clones were selected based on the suppressive effect of DEX on the proliferative responses of antigen-stimulated Th clones. Airway infiltration of eosinophils and lymphocytes of mice transferred with SS clones were effectively inhibited by the administration of DEX. In contrast, those of mice transferred with SR clones were not significantly inhibited. Administration of CTLA4-Ig significantly suppressed the proliferation of DEX-treated SR clones *in vitro*, and the eosinophil infiltration of mice transferred with SR clones *in vivo*.

## Conclusions

Steroid sensitivity of Th clones assessed *in vitro* was consistent with that of adoptively transferred asthma model assessed *in vivo*. Costimulatory signal mediated through CD28 is crucial for the induction of steroid resistance both *in vitro* and *in vivo*.

